# The Timing of Learning before Night-Time Sleep Differentially Affects Declarative and Procedural Long-Term Memory Consolidation in Adolescents

**DOI:** 10.1371/journal.pone.0040963

**Published:** 2012-07-12

**Authors:** Johannes Holz, Hannah Piosczyk, Nina Landmann, Bernd Feige, Kai Spiegelhalder, Dieter Riemann, Christoph Nissen, Ulrich Voderholzer

**Affiliations:** 1 Department of Psychiatry and Psychotherapy, University Medical Center Freiburg, Freiburg, Germany; 2 Schoen Clinic Roseneck, Prien am Chiemsee, Germany; Max Planck Institute of Psychiatry, Germany

## Abstract

Sleep after learning has been shown to foster the consolidation of new memories. However, fundamental questions on the best timing of learning before night-time sleep persist. We tested the hypothesis that learning directly prior to night-time sleep compared to 7.5 hrs prior to night-time sleep provides better conditions for the consolidation of declarative and procedural memories. Fifty healthy female adolescents (aged 16–17 years) were trained on a declarative word-pair and a procedural finger-tapping task at 3 pm (afternoon group, n = 25) or at 9 pm (evening group, n = 25), followed by a sleep laboratory night. Retrieval was assessed 24 hours and 7 days after initial training. Subjects trained in the afternoon showed a significantly elevated retention rate of word-pairs compared to subjects trained in the evening after 24 hours, but not after 7 days. In contrast, off-line gains in finger-tapping performance were significantly higher in subjects trained in the evening compared to those trained in the afternoon after both retention intervals. The observed enhanced consolidation of procedural memories after training in the evening fits to current models of sleep-related memory consolidation. In contrast, the higher retention of declarative memories after encoding in the afternoon is surprising, appeared to be less robust and needs further investigation.

## Introduction

Newly encoded memory traces have been shown to evolve in a critical time window after learning when they are still fragile and susceptible to disruptive stimulus interference [1]. Research indicates that periods of sleep occurring in this window can facilitate the process of memory consolidation (for review see [2]). However, it remains unclear whether the timing of learning prior to night-time sleep affects long-term memory consolidation [3]. This appears to be of particular relevance in adolescence since this developmental period is especially critical for learning [4], [5]. Thus, many countries have set a priority on further investigating the most favorable conditions for learning in children and adolescents (e.g., Program for International Student Assessment, PISA, [6]).

Adolescents in most developed countries tend to go to school in the morning, whereas activities in the late afternoon and evening are more flexible and partly characterized by the acquisition of novel memories, such as required for vocabulary learning or motor skills in sports and music. According to the most widely accepted classification scheme, these activities can be attributed to two major memory systems, the declarative and the procedural memory system [7].

Declarative memory consolidation refers to a process by which the newly encoded and initially instable memory traces of facts or knowledge, such as novel vocabulary, become stabilized and integrated into long-term representations [8], [9]. Animal and human studies [10] indicate that this consolidation depends on synaptic long-term plasticity in a hippocampal-neocortical network [11], [12]. In contrast, procedural memory is expressed as the, at least in part, implicit improvement of skills with practice, such as expert movements in sports or music. Procedural learning has been shown to be largely independent of the hippocampus, but to be mediated by the basal ganglia, the cerebellum and other brain structures [13]. Both memory systems have been demonstrated to differ in the type of the memory representation, the time course of acquisition and consolidation, and the underlying neural networks [7].

Over the past decades, a substantive body of work has been dedicated to the clinical [14] and molecular prerequisites of learning [1]. Particularly, periods of night-time sleep after learning have been shown to enhance the consolidation of declarative [12] and procedural memories [15] in comparison to equal periods of wakefulness. Current models propose that distinct components of sleep, such as sleep spindles [16] and electroencephalographic (EEG) slow wave activity during NREM sleep [17], might act in concert to strengthen and sharpen the novel memory representation. However, the best timing of acquisition for the consolidation of distinct types of memories remains largely unclear.

The aim of this study was to further determine the best timing of learning before night-time sleep for long-term memory consolidation in adolescents. We hypothesized that learning in the evening compared to learning in the afternoon would lead to better retrieval performance on a declarative and a procedural memory task. Thus, we trained 50 adolescents either at 3 pm in the afternoon, 7.5 hours before night-time sleep, or in the evening at 9 pm, directly before night-time sleep, on a declarative (associate word-pairs) and procedural (finger-tapping) task and assessed memory consolidation across a retention interval of 24 hours and 7 days after initial acquisition.

## Results

**Figure 1:**
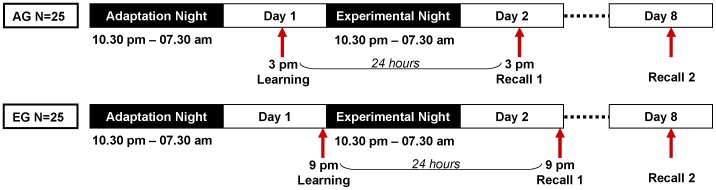
Study design. In the afternoon group, 25 subjects were trained at 3 pm, followed by standardized activities (e.g., card games, movies). In the evening group, 25 subjects were trained at 9 pm. The experimental sleep-laboratory night (10:30 pm to 7:30 am) followed a first adaptation night in the sleep laboratory. Parameters of memory consolidation were assessed across retention intervals of 24 hours and 7 days.

Fifty adolescent female subjects (aged 16–17 years) were randomly assigned to one of two experimental groups. Twenty-five subjects were trained at 3 pm (afternoon group, AG), 25 subjects were trained at 9 pm (evening group, EG), followed by a sleep laboratory night from 10.30 pm to 7.30 am. Parameters of memory consolidation were assessed across retention intervals of 24 hours and 7 days. Figure 1 summarizes the experimental design.

### Declarative Word-Pair Task

To test *declarative memory consolidation*, a word-pair association task was used in an adapted version consisting of 46 semantically related word pairs [18]. Memory consolidation was calculated as the percentage of correctly retrieved words at recall referred to the number of correctly encoded words at baseline (retention rate, %) (please refer to the Materials and Methods section for more details).

At initial encoding at baseline (Day 1), the experimental groups did not differ in the number of trials to criterion or in the final number of correctly encoded word-pairs (Table 1). An analysis of variance (ANOVA) with the repeated-measures factor Time (Day 1, 2 and 8) and the between-subject factor Group (AG, EG) showed a highly significant Time effect (*F* = 22.6, *P*<0.001), no significant Group effect (*F* = 0.1, *P* = 0.772) and a significant Time X Group interaction (*F* = 3.3, *P* = 0.040) for the number of correctly retrieved word-pairs, indicative for significant differences in the time course of declarative memory consolidation between the groups.

**Table 1:** Results declarative word-pair task.

Afternoon group n = 25		Evening group n = 25	*F*	*P*
**Word pair performance on Day 1**				
** N° of trials to criterion**	1.3±0.5	1.5±0.5	3.1	0.086
** N° of retrieved word pairs**	35.0±4.7	36.4±4.3	1.1	0.304
**Retention rate referred to Day 1**				
** Word pair retention rate on Day 2, %**	98.7±6.6	95.0±3.5	6.1	**0.016**
** Word pair retention rate on Day 8, %**	96.1±5.7	92.1±5.6	3.9	0.053

Values represent means ± SD. N°, number. Statistics refer to direct comparisons between the afternoon and evening group. Significant values are given in bold, *P*<0.05.

**Figure 2:**
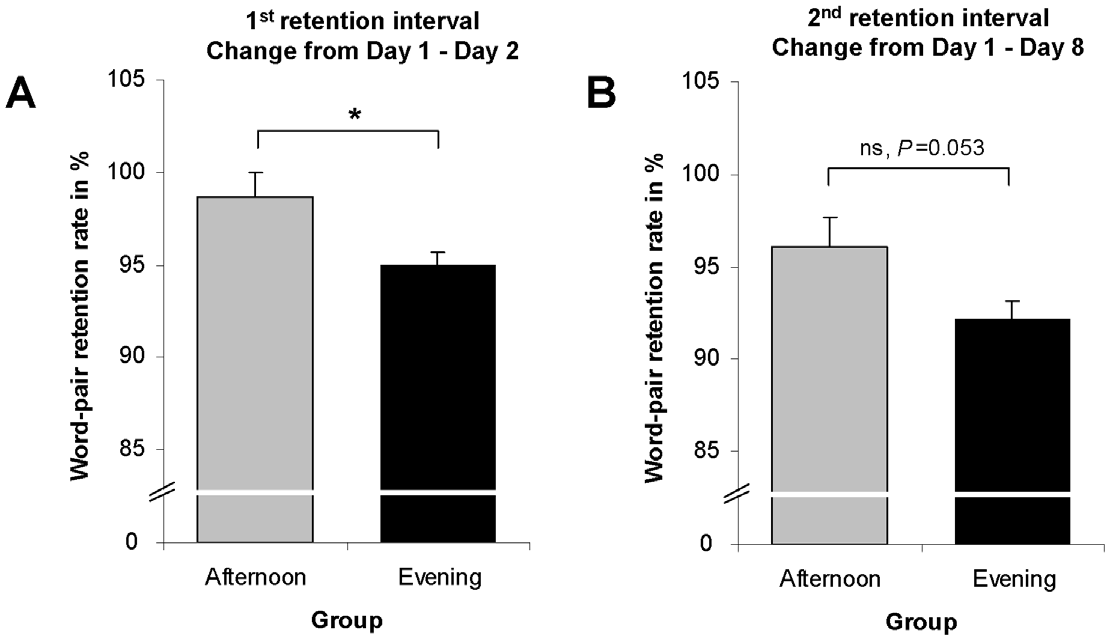
Declarative word-pair task. A. Subjects in the afternoon group showed a significantly higher retention rate of correctly recalled word-pairs in the retention interval from Day 1 to Day 2 compared to the evening group. B. In the retention interval from Day 1 to Day 8, the difference tended to persist but slightly failed to reach statistical significance (*P = *0.053). Error bars depict SE. The asterisk (*) indicates statistical significance, *P*<0.05.

To further specify the direction of this interaction, we calculated and compared the retention rates between both groups (percentage of correctly retrieved word-pairs at recall relative to the number of correctly retrieved word-pairs at baseline) for both retention intervals separately (Tab. 1). As the first main result, this analysis revealed a significantly elevated retention rate from Day 1 to Day 2 in the AG compared to the EG (Tab. 1, Fig. 2a). The difference between the groups in the retention interval from Day 1 to Day 8 slightly failed to reach statistical significance (Tab. 1, Fig. 2b).

### Finger Tapping Task

To test *procedural memory consolidation* we used a finger-tapping task adopted from previous studies [19]. This task required the subjects to repeatedly tap a five-element sequence of numbers (4–2–3–1–4) on a keyboard as fast and accurately as possible (please refer to the Materials and Methods section for details).

At initial training at baseline (Day 1), the experimental groups did not differ in the finger-tapping speed (number of correctly completed sequences) or accuracy (error rate, defined as the number of errors relative to the total number of tapped sequences in percent). The data of the procedural finger-tapping task are summarized in Table 2.

**Table 2:** Results procedural finger-tapping task.

Afternoon group n = 25		Evening group n = 25	*F*	*P*
**FT performance on Day 1**				
** Speed (N° of correct sequences)**	14.4±2.7	14.9±3.8	0.3	0.582
** Accuracy (error rate, %)**	6.9±4.4	9.5±5.5	3.6	0.064
**Improvement in FT speed and accuracy referred to Day 1**				
** Difference in FT speed on Day 2**	4.8±1.7	6.1±1.8	5.8	**0.020**
** Difference in FT speed on Day 8**	6.8±2.3	9.0±2.4	10.6	**0.002**
** Difference in accuracy on Day 2**	0.1±4.1	−3.1±5.3	5.6	**0.022**
** Difference in accuracy on Day 8**	0.3±4.3	−3.7±5.8	8.3	**0.006**

Values represent means ± SD. FT, finger tapping. N°, number. Statistics refer to direct comparisons between the afternoon and evening group. Significant values are given in bold, *P*<0.05.

An ANOVA with the repeated-measures factor Time (Day 1, 2 and 8) and the between-subject factor Group (AG, EG) showed an overall improvement in finger-tapping performance (multivariate effect for the factor Time: *F* = 144.7, *P<*0.001), no significant multivariate Group effect (*F* = 1.5, *P = *0.220) and a highly significant Group X Time interaction (speed: *F = *7.8, *P = *0.001; accuracy: *F = *5.5, *P* = 0.006).

**Figure 3:**
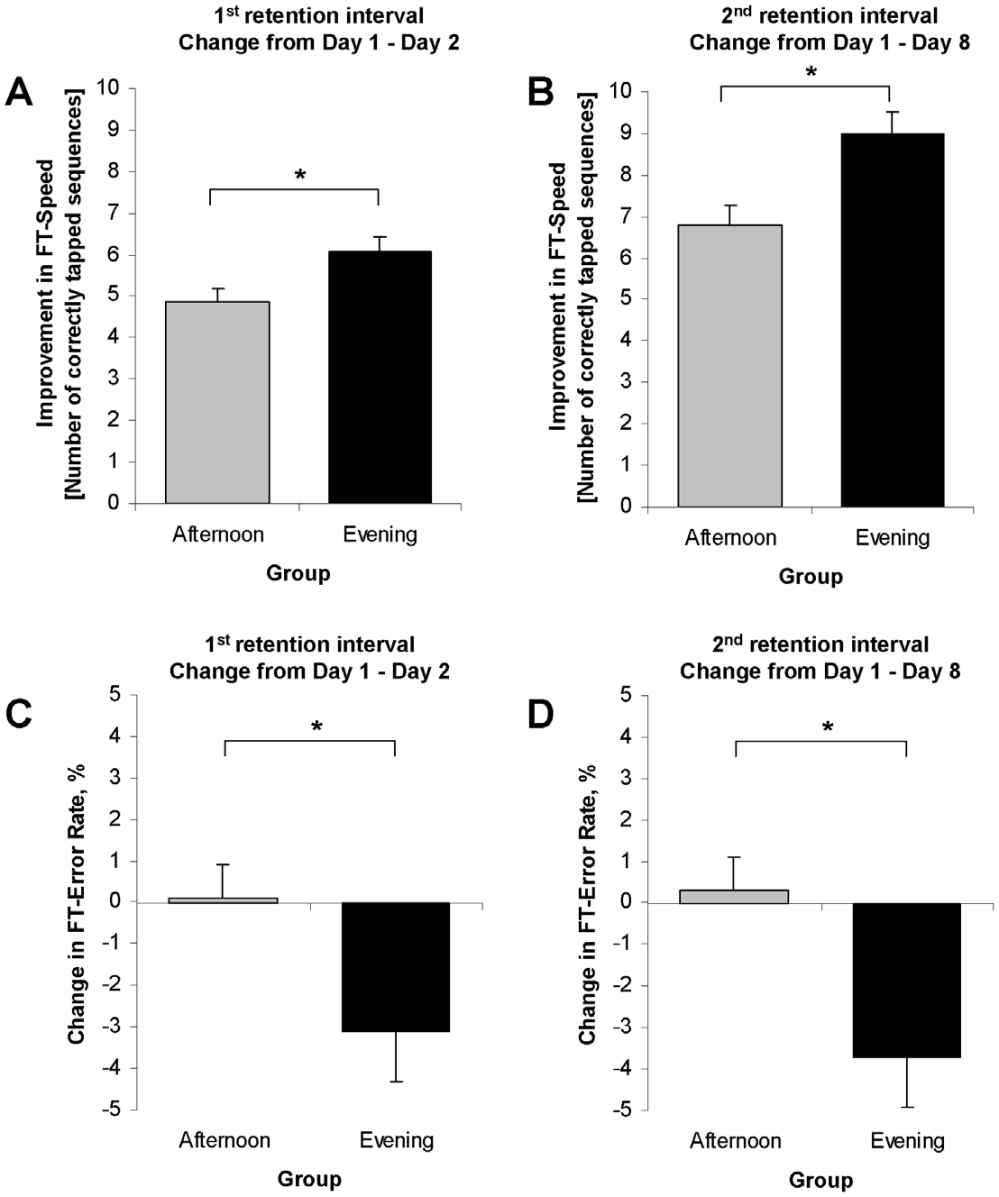
Procedural finger-tapping task. **A/B.** Subjects in the evening group showed a significantly greater improvement in finger-tapping speed across the first (**A**) and second (**B**) retention interval compared to the afternoon group**. C/D.** Subjects in the evening group showed a greater gain in accuracy (as indexed by a decrease in the error rate) across the first (**C**) and second retention interval (**D**)**.** Error bars depict SE, the asterisks (*) indicate statistical significance, *P*<0.05.

To further specify the direction of the observed Group X Time interaction, we calculated and compared the change in performance for both retention intervals separately (Tab. 2). Univariate ANOVAs revealed a significantly greater improvement in finger-tapping speed in the EG compared to the AG across the first retention interval from Day 1 to Day 2 (Tab. 2, Fig. 3a), as well as for the interval from Day 1 to Day 8 (Tab. 2, Fig. 3b). Furthermore, the analysis also revealed a greater improvement in accuracy in the EG compared to the AG for the retention interval from Day 1 to Day 2 (Tab. 2, Fig. 3c), as well as for the interval from Day 1 to Day 8 (Fig. 3d).

**Figure 4:**
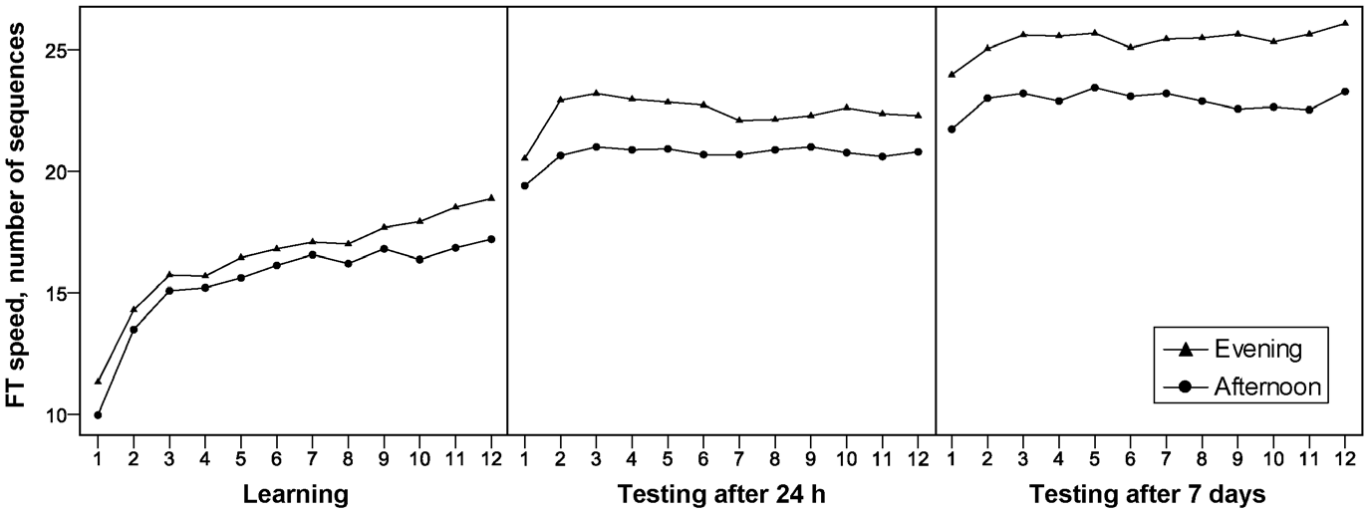
Time course of finger tapping speed across the baseline training session, first (24 hrs) and second (7 days) test session. As depicted, the evening group showed higher off-line gains in performance from the first to the second as well as from the second to the third test session compared to the afternoon group.

To check whether the reported differences between the learning and retrieval sessions were caused by off-line changes and not merely by changes in within-session performance, we analyzed the mean difference between the values of every single trial of a session and the first (baseline) value of the same session in a repeated measures ANOVA with the repeated-measure factor Time (Day 1, 2 and 8) and the between-subject factor Group (AG, EG). There were no significant effects for the factors Time, Group and Group X Time interaction (all *Ps*>0.05). This indicates that the observed group differences in between test-session changes cannot be explained by differences in within-session changes. Figure 4 illustrates the time course of finger-tapping speed across all test sessions.

### Sleep Recordings

Table 3 summarizes the polysomnographic parameters for both experimental groups. Analyses revealed normal sleep data and no significant differences in any of the sleep parameters between the groups. Analyses of the association between polysomnographic parameters and measures of memory consolidation in the experimental groups did not reveal any significant correlation (*P*>0.05, results not shown). To further address the question whether the slight differences in sleep parameters might have driven the results, we repeated our analysis including sleep latency, slow wave sleep (% SPT) and REM sleep (% SPT) as covariates into the statistical model. Most importantly, for both memory tasks none of the covariates reached statistical significance, neither for the first retention interval (Day 1 to Day 2; *Ps>*0.05) nor for the second (Day 1 to Day 8; *Ps*>0.05). Nevertheless we calculated the ANOVAs including sleep latency, slow wave sleep (% SPT) and REM sleep (% SPT) as covariates. The differences in word-pair retention remained significant between the experimental groups for the covariate sleep latency (F = 4.9, *P* = 0.032) and slow wave sleep (% SPT) (F = 4.4, *P* = 0.040), but slightly dropped under the level of statistical significance when entering REM sleep (% SPT) as a covariate (F = 4.0, *P* = 0.053). The results of the finger-tapping task remained significant between the experimental groups for the covariate sleep latency (FT speed: F = 4.5, *P* = 0.039; FT accuracy: F = 6.9, *P* = 0.012), slow wave sleep (% SPT) (FT speed: F = 4.7, *P* = 0.035; FT accuracy: F = 6.6, *P* = 0.014) and REM sleep (% SPT) when looking at FT accuracy (F = 5.3, *P* = 0.026), but slightly dropped under the level of statistical significance when looking at FT speed (F = 3.9, *P* = 0.054).

**Table 3:** Polysomnographic sleep parameters.

	Afternoon group n = 25	Evening group n = 25	*F*	*P*
**Sleep latency, min**	18.5±9.3	26.0±17.9	3.4	0.072
**Sleep period time, min**	517.5±12.6	511.2±17.9	2.1	0.154
**Sleep efficiency, %**	92.0±3.5	90.4±4.7	1.9	0.172
**Waking (% SPT)**	4.0±2.1	4.5±3.5	0.4	0.528
**Stage 1 (% SPT)**	4.2±1.7	4.4±2.5	0.1	0.711
**Stage 2 (% SPT)**	46.3±5.9	44.0±6.6	1.6	0.208
**Slow wave sleep (% SPT)**	23.8±6.6	27.3±8.2	2.8	0.099
**REM sleep (% SPT)**	21.8±3.9	19.8±3.8	3.3	0.075

Values represent means ± SD. REM, rapid eye movement; SPT, sleep period time.

### Control Variables

All participants reported a high subjective sleep quality and no signs for depression (Tab. 4). They had normal levels of perceived stress and a high motivation for learning (Scales to Asses Goal Orientation and Achievement Motivation, [20]). As listed in Table 4, the experimental groups did not differ in any of these parameters.

**Table 4:** Study sample.

	Afternoon group n = 25	Evening group n = 25	*F*	*P*
**Age in years**	16.7±0.5	16.5±0.5	1.3	0.257
**IQ (SPM)**	103.4±9.3	104.1±9.1	0.1	0.807
**BDI**	3.3±3.1	3.6±3.3	1.3	0.720
**PSQ-20**	28.2±13.8	23.3±13.5	1.6	0.215
**SELLMO – Achievement motivation**	55.2±13.4	57.8±9.1	0.6	0.425
**PSQI – Total score**	3.4±1.3	3.0±1.6	1.0	0.332
**PSQI – Habitual sleep time**	7.9±0.6	8.0±0.7	0.1	0.705
**SF-A sleep quality**	3.9±0.6	4.0±0.5	0.2	0.663
**ESS**	6.2±2.8	6.7±2.9	0.4	0.534
**TAP visual alertness (ms)**	256.7±26.9	243.9±27.7	2.6	0.111
**TAP auditory alertness (ms)**	260.7±28.3	248.0±30.0	2.3	0.140
**Working memory (digit span test)**	16.2±3.1	16.5±3.0	0.1	0.746

Values represent means ± SD. IQ, Intelligence quotient; SPM, Standard Progressive Matrices; BDI, Beck Depression Inventory; PSQ-20, Perceived Stress Questionnaire 20 item version; SELLMO, Scales to Asses Goal Orientation and Achievement Motivation; PSQI, Pittsburgh Sleep Quality Index; SF-A, Sleep Questionnaire A; ESS, Epworth Sleepiness Scale; TAP, Testbattery for the Assessment of Attention.

Additional neuropsychological assessments (measures of alertness and working memory) prior to the test sessions did not reveal performance differences between the two experimental groups (*P*>0.05, Tab. 4). This suggests that the effects observed for declarative and procedural memory consolidation can not be explained by differences in alertness or working memory.

## Discussion

The present findings propose a new aspect of effective learning strategies for adolescents. Based on studies of overnight memory consolidation, we demonstrated that off-line gains in procedural finger-tapping were significantly increased in subjects trained in the evening directly before night-time sleep compared to subjects trained in the afternoon 7.5 hrs before night-time sleep. In contrast, declarative memories for word-pairs were preferentially retrieved after encoding in the afternoon compared to encoding in the evening.

Recent studies have shown that sleep after learning facilitates the consolidation of novel procedural and declarative memories in comparison to equal periods of wakefulness (for review see [2]). Other studies have shown that these processes are, at least in part, impaired in patients with primary sleep disorders [21], [22]. Our study extends the concept of sleep-related memory consolidation by providing first evidence that the timing of learning before night-time sleep differentially affects the consolidation processes in the two major memory systems.

Our observation that motor learning directly before night-time sleep was especially effective fits to current models of a time- and sleep-dependent process of motor skill consolidation (for review see [23]). The effect of sleep on motor learning has been demonstrated to be particularly high within the first 4–6 hrs after initial training – a period of enhanced susceptibility of initially instable procedural memory representations to disruptive interference [24]. Furthermore, the consolidation of a new motor skill has been shown to be impaired by subsequent training of a second skill, but no disruption was observed if at least 4 hrs elapsed between the training sessions [25], [26]. Consistently, Korman et al. [27] showed that the robustness to interference of a finger-opposition tapping task 2 hrs after training was significantly enhanced when subjects slept 90 min immediately after training. Sleep-specific brain activity patterns, such as sleep spindles [28] or electroencephalographic slow wave activity [17], might foster this consolidation process in the procedural memory system [29]. Preclinical studies have provided support for a time- and sleep-dependent consolidation process. On a cellular level, de novo protein synthesis and gene expression after training seem to be necessary for successful motor skill learning [30], [31] and have been shown to be modified during subsequent sleep [32]–[34]. Taken together, these studies support our finding that procedural motor consolidation is especially effective when training takes place in the evening, directly prior to night-time sleep.

Our second main finding – the preferential overnight retention of declarative memories after encoding in the afternoon – contradicts previous studies on sleep-related memory consolidation. Specifically, Gais et al. [35] reported enhanced declarative memory consolidation of vocabulary words in adolescents when sleep directly followed initial acquisition. Similarly, Talamini et al. [36] showed in adults that the consolidation of declarative associative (spatial) memories was significantly higher when sleep directly followed the initial training session. These studies focused on the effects of sleep directly after learning in comparison to waking conditions with sleep delayed for longer periods of 11 hrs (Talamini et al.) and 15 hrs (Gais et al.) where learning took place in the morning or evening. Differences in homeostatic aspects (shorter interval of 7.5 hrs between acquisition and night-time sleep) and the circadian phase (acquisition in the afternoon) in the present study might explain the different effect on the development of declarative memory traces. A possible molecular mechanism for the enhanced retention after early encoding observed in the current study arises from preclinical studies showing that the time course of declarative memory is strongly dependent on the late phase of hippocampal long-term potentiation (LTP) which is known to last from 3 to 24 hrs after encoding [37]. The late phase of LTP requires both gene transcription and translation, leading to the growth of new synaptic connections [38] and is known to benefit from post-learning sleep [39], [40]. Thus, intense declarative learning in the afternoon might allow for pre-sleep processes of plasticity and for the coincidence of night-time sleep with a critical window of synaptic long-term plasticity in a hippocampal-neocortical network required for declarative memory consolidation.

Further analyses of our declarative findings revealed that the preferential retention of declarative memories after encoding in the afternoon was not sustained across a retention interval of 7 days (*P* = 0.053). Furthermore, we can not exclude the possibility that slight but non-significant differences in sleep parameters between the groups might have contributed to the results. Thus, the declarative findings appear less robust than the procedural results and should be interpreted with caution.

From a translational perspective, our results may have relevant implications: it might be more effective to learn declarative material, like vocabulary words, in the afternoon and to train procedural skills, such as those required for music or sports, in the evening. Remarkably, training in the evening, compared to training in the afternoon, resulted in a significantly elevated gain in motor performance not only 24 hrs after initial training, but also at follow-up after one week. As noted earlier, our declarative findings appear less robust and should be interpreted with caution. Together, our results are informative for the development of new and potentially more effective teaching and learning strategies for adolescents, their parents and teachers.

Whereas we observed robust effects on the behavioral level, relevant questions on the potential mechanisms persist. First, it is possible that the observed effects were not driven by sleep-related factors, but result, at least in part, from differences in the circadian phase of the encoding and retrieval sessions (afternoon group 3 pm, evening group 9 pm). In our study, baseline parameters for acquisition, attention and working-memory were similar in both experimental groups. However, to further disentangle sleep-dependent from circadian effects, it would be necessary to additionally study an afternoon and evening control group under conditions of total sleep deprivation. Second, differences in memory consolidation might arise from different levels of stimulus interference across the retention interval. Importantly, in our study, retrieval was assessed 24 hours and 7 days after acquisition in both the afternoon and the evening group and the level of interference was kept comparable during wakefulness. Thus, the timing of sleep or the circadian phase rather than differences in interference explain our findings. It is important to note, however, that different aspects of the memory tasks itself can have interfering effects on the consolidation in the respective memory system. There is some evidence showing that reciprocal interactions between memory systems might occur during wakefulness but might be processed independently during sleep [41], [42]. Finally, one should bear in mind that our findings are restricted to female adolescents. All participants demonstrated post-pubertal status and were strictly investigated in their follicular phase of the menstrual cycle. Whether our findings might translate to male adolescents and potentially also to adults remains unclear. Additional studies are needed to investigate the effects of the timing of learning before night-time sleep on memory consolidation in these populations.

In conclusion, our results indicate that learning directly before night-time sleep preferentially promotes procedural memory consolidation, whereas – with less confidence – learning in the afternoon, 7.5 h before night-time sleep, might provide better conditions for the consolidation of declarative memories in adolescents. Even though it should be borne in mind that this is the first study showing these results, the findings might contribute to the development of new effective teaching and learning strategies. Translating the results to the every-day life of adolescents, we propose that declarative memories, such as vocabulary words, should be studied in the afternoon and motor skills, like playing soccer or piano, should be trained in the late evening. Most parents among us would have preferred the opposite results.

## Materials and Methods

### Participants

Of N = 64 screened subjects, 8 subjects did not meet inclusion criteria (3 psychiatric history, 2 sleep disturbances, 2 amenorrhea, 1 not interested in participation). Due to technical problems, finger tapping data were lost for 6 subjects. The analyses are based on a final sample of N = 50 subjects (all female, aged 16–17 years). Screening assessments included a clinical interview, physical examination, routine blood work, urine drug screen, and self report measures to rule out any physical or mental disorders. General intelligence was assessed using the Standard Progressive Matrices (SPM, [43]). Good sleeper status was demonstrated by completion of the Pittsburgh Sleep Quality Index (PSQI, [44], cut-off >5) and the Epworth Sleepiness Scale (ESS, [45]). Tab. 4 summarizes the description of the sample.

All participants followed a regular sleep-wake pattern with an average sleep time of about 8 h per night within the week prior to and during the study, as determined by sleep diaries and actigraphy [46]. All subjects were right handed (Edinburgh Handedness Inventory [47]) and demonstrated normal or corrected-to-normal vision. Importantly, all subjects demonstrated late-pubertal status according to the rating scale for pubertal development [48] and were in the follicular phase of their menstrual cycle. All subjects were free of any medication or substance use. The restricted range of sex, age and school type was chosen to reduce variance related to the potential effects of developmental aspects and the level of education. Subjects were recruited from local secondary schools in the area of Freiburg and received financial reimbursement for participation. Written informed consent was obtained from all participants and their parents (or legal representatives) prior to the onset of the study. The procedures had been approved by the local institutional review board (EK 328/08) and carried out according to the Declaration of Helsinki.

### Study Design

To ensure well controlled experimental conditions, subjects were randomized to one of the two experimental groups (parallel group design). The strict inclusion criteria ensured that the study sample was highly homogenous in demographical parameters. Figure 1 summarizes the experimental design. In the afternoon group (AG), subjects completed a learning session in the afternoon (3 pm). Then, they underwent supervised and standardized activities (e.g., card games, movie, dinner, preparation for PSG recordings) and went to bed at 10.30 pm. In the evening group (EG), subjects completed the learning session at 9 pm, directly before going to bed. The experimental sleep-laboratory night (10:30 pm to 7:30 am) followed an adaptation night that served as adaptation to the sleep laboratory conditions and was used to rule out any sleep abnormalities. To keep the time interval and the amount of interference comparable, retrieval was assessed 24 hrs after initial acquisition in both groups (AG 3 pm, EG 9 pm). To test for long-term effects, retrieval was re-assessed 7 days later at 3 pm.

### Declarative Word-Pair Task

**Figure 5:**
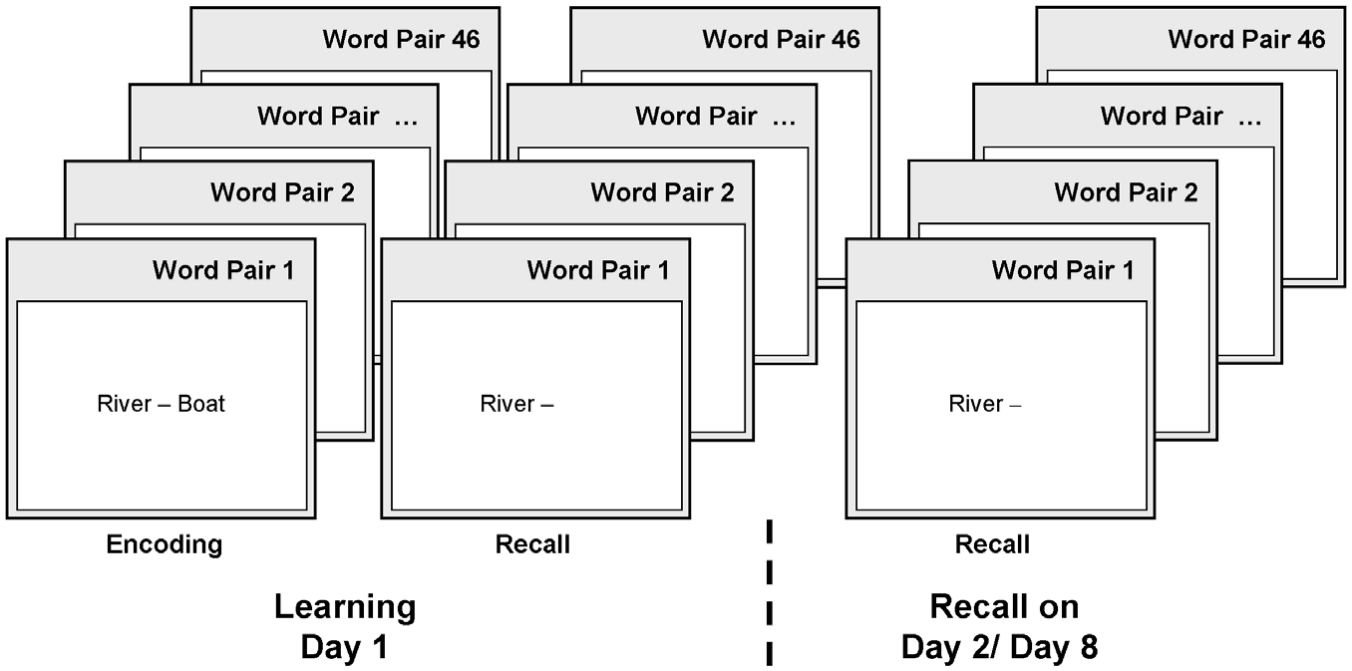
Word-pair association task. To test *declarative memory consolidation*, the word-pair association task was used in an adapted version consisting of 46 related word pairs presented randomly on a 15 inch computer screen for 5000 ms, followed by a 100 ms blank screen using the Presentation® software (word-pair list and procedures were identical to the ones used by [18]). Four additional word pairs at the beginning and end of the task served to buffer primacy and recency effects. The word pairs were presented repeatedly until the subject remembered at least 60% in a cued-recall test, i.e. stating the word matching the first word of the previously learned word pairs. Variables measuring declarative memory encoding on day 1 (baseline) were the number of trials to criterion as well as the number of correctly retrieved word pairs in the last trial. Memory consolidation was calculated as the percentage of correctly retrieved words at recall referred to the number of correctly encoded words in the learning session (retention rate, %). Cued-recall was performed one single time without further learning 24 hrs and 7 days after initial acquisition and the number of correctly recalled words was assessed.

To test *declarative memory consolidation*, the word-pair association task was used in an adapted version consisting of 46 semantically related word pairs (Fig. 5). The word pairs were presented repeatedly until the subject remembered at least 60% in a cued-recall test, i.e. stating the word matching the first word of the previously learned word pairs. At baseline, performance was assessed as the number of correctly retrieved word-pairs in the last learning trial as well as the number of trials to criterion. Cued-recall was performed one single time without further learning 24 hrs and 7 days after initial learning. Memory consolidation was calculated as the percentage of correctly retrieved word-pairs at recall relative to the number of correctly retrieved word-pairs at baseline (retention rate, %).

### Procedural Finger-Tapping Task

**Figure 6:**
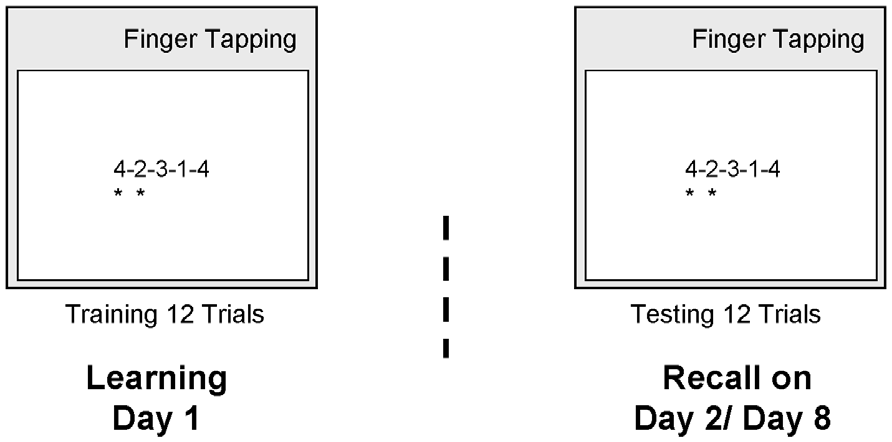
Procedural finger-tapping task. The task required the subjects to repeatedly tap a five-element number sequence (4–2–3–1–4) on a keyboard with the fingers of the non-dominant hand as fast and accurately as possible for 30-s epochs interrupted by 30-s breaks. To keep working memory load at a minimum, the numeric sequence was always displayed on the computer screen while tapping. A key press resulted in an asterisk displayed under the corresponding numeric character. For each 30-s trial the speed (number of correctly completed sequences) and accuracy (error rate, defined as the number of errors relative to the total number of tapped sequences in percent) was calculated. To reinforce optimal performance, the speed and error rate were displayed to the subjects after each 30-s trial. To familiarize participants with the task, five sequences were performed prior to the test session (not included in the analysis). Each test session consisted of twelve 30-s trials. The level of performance for each session was calculated as the averaged speed and accuracy across all trials.

The finger-tapping task [19] has been adopted from previous studies indicating robust sleep-related improvement on this task. The software version was provided by Dr. Rasch [49]. It required the subjects to repeatedly tap a five-element number sequence (4–2–3–1–4) on a keyboard as fast and accurately as possible (Fig. 6). Each test session consisted of twelve 30-s trials. The level of performance for each session was calculated as the averaged speed and accuracy across all trials.

### Sleep Recordings

Polysomnography was recorded during the two sleep laboratory nights (adaptation and experimental night) according to standardized procedures [50]. The setup included the electroencephalographic (EEG) electrodes C3–A2 and C4–A1, submental electromyogram (EMG), vertical and horizontal electrooculogram (EOG) and electrocardiogram (ECG). Sleep recordings were scored visually by experienced raters using standard criteria [51]. The following variables of sleep continuity and architecture were assessed: sleep period time (defined as the period between sleep onset and final awakening), sleep efficiency (defined as the ratio of total sleep time to time in bed * 100%), sleep latency (defined as the period between turning the lights off and the first 30-second epoch of sleep), as well as the time spent in waking and in the sleep stages 1, 2, slow wave sleep (SWS, combined stages 3 and 4), and REM sleep (expressed as percentage of sleep period time).

### Neuropsychological Assessments

Cognitive performance, including alertness (Test for Attentional Performance [52]) and working memory (Digit Span and Block Tapping [53]), was assessed prior to each test session to control for confounding effects of cognitive functioning.

### Data Analysis

Data was analyzed using the Predictive Analytics Software (PASW) version 18.0 (SPSS Inc., Chicago, IL, USA). Means and standard deviations were calculated for descriptive purposes. One-way analyses of variance (ANOVA) were used to test for group differences in demographic characteristics, polysomnographic parameters, subjective sleep parameters, and baseline memory performance. To compare memory consolidation, a 3×2 ANOVA with the repeated-measurement factor Time (Day 1, Day 2 and Day 8) and the between subject factor Group (AG, EG) was calculated. To further specify the time course of memory consolidation between the groups, we calculated and tested the change in memory performance for the first (Day 1 to retrieval on Day 2) and second retrieval interval (Day 1 to retrieval on Day 8) separately. To check whether the reported differences between the learning and retrieval sessions were caused by off-line changes and not merely by changes in within-session performance, we zeroized each test session by subtracting the mean difference between the values of every single trial of a session and the first (baseline) value of the same session. The values were then averaged across test sessions and analyzed in a repeated measures ANOVA with the repeated-measure factor Time (Day 1, 2 and 8) and the between-subject factor Group (AG, EG) to test for within-session changes. Bivariate Pearson correlation analyses were used to investigate the relationships between performance in the memory tasks and polysomnographic sleep parameters. The level of significance was set at *P*<.05 (two-tailed).
